# Living at the farm, innovative nursing home care for people with dementia – study protocol of an observational longitudinal study

**DOI:** 10.1186/s12877-015-0141-x

**Published:** 2015-11-02

**Authors:** B. de Boer, J.P.H. Hamers, H.C. Beerens, S.M.G. Zwakhalen, F.E.S. Tan, H. Verbeek

**Affiliations:** Department of Health Services Research, Faculty of Health, Medicine and Life Sciences, CAPHRI School for Public Health and Primary Care, Maastricht University, Maastricht, The Netherlands; Department of Methodology and Statistics, Faculty of Health, Medicine and Life Sciences, CAPHRI School for Public Health and Primary Care, Maastricht University, Maastricht, The Netherlands

**Keywords:** Activities, Daily life, Dementia, Green care farms, Institutional long-term care, Nursing home care environments, Quality of care, Quality of life, Social engagement

## Abstract

**Background:**

In nursing home care, new care environments directed towards small-scale and homelike environments are developing. The green care farm, which provides 24-h nursing home care for people with dementia, is one such new care environment. Knowledge is needed on the relation between environmental features of green care farms such as nature, domesticity and offering care in small groups and the influence on the daily lives of residents. The aim of this study is to explore (1) the daily lives of residents, (2) the quality of care and (3) the experiences of caregivers on green care farms compared with other nursing home care environments.

**Methods/design:**

An observational longitudinal study including a baseline and a six-month follow-up measurement is carried out. Four types of nursing home care environments are included: (1) large scale nursing home ward, (2) small scale living facility on the terrain of a larger nursing home (3) stand-alone small scale living facility and (4) green care farm. Quality of care is examined through structure, process and outcome indicators. The primary outcome measure is the daily life of residents, assessed by ecological momentary assessments. Aspects of daily life include (1) activity (activity performed by the resident, the engagement in this activity and the degree of physical effort); (2) physical environment (the location of the resident and the interaction with the physical environment); (3) social environment (the level and type of social interaction, and with whom this social interaction took place) and (4) psychological well-being (mood and agitation). In addition, social engagement, quality of life, behavioral symptoms and agitation are evaluated through questionnaires. Furthermore, demographics, cognitive impairment, functional dependence and the severity of dementia are assessed. Semi-structured interviews are performed with caregivers regarding their experiences with the different nursing home care environments.

**Discussion:**

This is the first study investigating green care farms providing 24-h nursing home care for people with dementia. The study provides valuable insight into the daily lives of residents, the quality of care, and the experiences of caregivers at green care farms in comparison with other nursing home care environments including small-scale care environments and large scale nursing home wards.

## Background

The number of people living with dementia worldwide is expected to increase from 24 million in 2001 to 81 million in 2040 [1]. This trend can also be seen in the Netherlands where the number of people suffering from dementia is expected to double to over 500,000 people in 2040 [2]. Most of the people with dementia live at home. However, as the dementia progresses, living at home is often not possible and approximately 30 % get admitted to nursing homes as they require complex care [3, 4].

Nursing home care used to be primarily organized according to a medical care concept [5–7] in traditional large-scale wards with an institutional character. Physical care needs were the main focus of attention and care for people with dementia was organized around routines of the nursing staff [8, 9]. In many countries, current nursing homes are increasingly organized according to a psychosocial and more homelike care concept [10, 11]. Here, the care is often organized in smaller units, usually with 6–8 residents [8]. The residents live together in a homelike and recognizable environment in which striving to achieve a situation closest to home is the priority [12, 13]. Personal care and daily routines are integrated, meaning that care staff performs tasks such as cooking and cleaning together with the residents. This psychosocial care concept strives to allow people to continue the life they had before admission, as much as possible, and promotes person centered care and quality of life. In addition, this type of care involves the provision of autonomy for residents, letting them make their own choices and encouraging social interaction and participation in activities [14, 15].

The change in care concept and the exponential growth of the number of people with dementia lead to a high demand for a broad selection of innovative and efficient nursing home care facilities that meet individuals’ desires and requirements [1]. In the Netherlands, a variety of small scale, homelike living facilities have been developed; some are stand-alone units in the neighborhood built as an archetypical house while others are units situated on the terrain of a larger nursing home [16]. New type of small-scale, homelike care facilities are green care farms for people with dementia. Originally developed as day-care only centers, nowadays some green care farms have started to provide 24-h care as a new alternative for regular nursing homes.

### What are green care farms?

Green care farms come in different forms, and are referred to in a variety of terms, such as social farming, multifunctional agriculture and farming for health [17]. The development of green care farms not only stems from the health care sector, but also from developments within the agricultural sector [18]. Here, there is an increasing demand for multifunctional agriculture in order to increase revenue for farmers [19]. Green care farms mostly provide day care for different client groups (e.g., people with learning disabilities, psychological problems, addiction problems and people with dementia) and are aimed at promoting individual's social, mental, and educational wellbeing [18, 20–22]. Green care farms that provide day care are developing in many parts of the world [19]. Leading countries include: Norway (approximately 1,100 green care farms), the Netherlands (1,000), France (900), Italy (675), Belgium (300), Austria (250), the UK (230), Germany (160) and Ireland (100) [17–19, 23]. Some of these green care farms are actual farms that have agricultural production while for others, providing care is the main source of income and gaining agricultural production is a byproduct [24].

In the Netherlands, approximately 200 green care farms provide day care for people with dementia [21]. Findings of a study investigating the effects of day care on Dutch green care farms suggest that there are differences in the daily lives between people spending their days at green care farms and those who spend their days at other day care facilities. Positive indications such as more frequently available activities and more variation in activities at green care farms were found [25]. In addition, research findings suggest higher involvement in activities of daily living (ADL) [26], and more physical effort needed [25] for people with dementia spending their daytime on green care farms. These findings are important because being engaged in activities allows people with dementia to connect with other people and to experience feelings of pleasure [27, 28]. Furthermore, participation in activities is associated with a higher quality of life [29] and is one of the priorities in nursing homes [30]. Other aspects related to the daily lives of people with dementia such as social relations, interaction with the physical environment and mood can also influence quality of life [31–33].

Recently, some green care farms that provide 24-h nursing home care are being developed. These green care farms have many characteristics of small scale living, meaning that a small group of residents live together in a home-like and non-institutional ‘house’ on the terrain of a farm. People living at green care farms have the opportunity to go outdoors and to take care of gardens or animals. Other daily activities include domestic activities (e.g., preparing dinner, dishwashing), work-related activities (e.g., cleaning the stables, feeding animals), social activities (e.g., coffee break, dinner) and leisure/recreational activities (e.g., playing a game, listening to music). This wide range of activities is incorporated into normal daily life activities [22].

More insight is needed into the added value of green care farms as they relate to the spectrum of nursing home care environments. The current study investigates (1) the daily lives of residents, (2) the quality of care, and (3) the experiences caregivers with the care environment. Green care farms are compared with of other forms of small-scale, homelike care environments and other regular large scale nursing home wards.

## Methods

### Design

This study uses an observational longitudinal design including a baseline measurement and a six-month follow-up measurement. The study takes place between April 2014 and December 2015.

### Setting

**Fig. 1:**
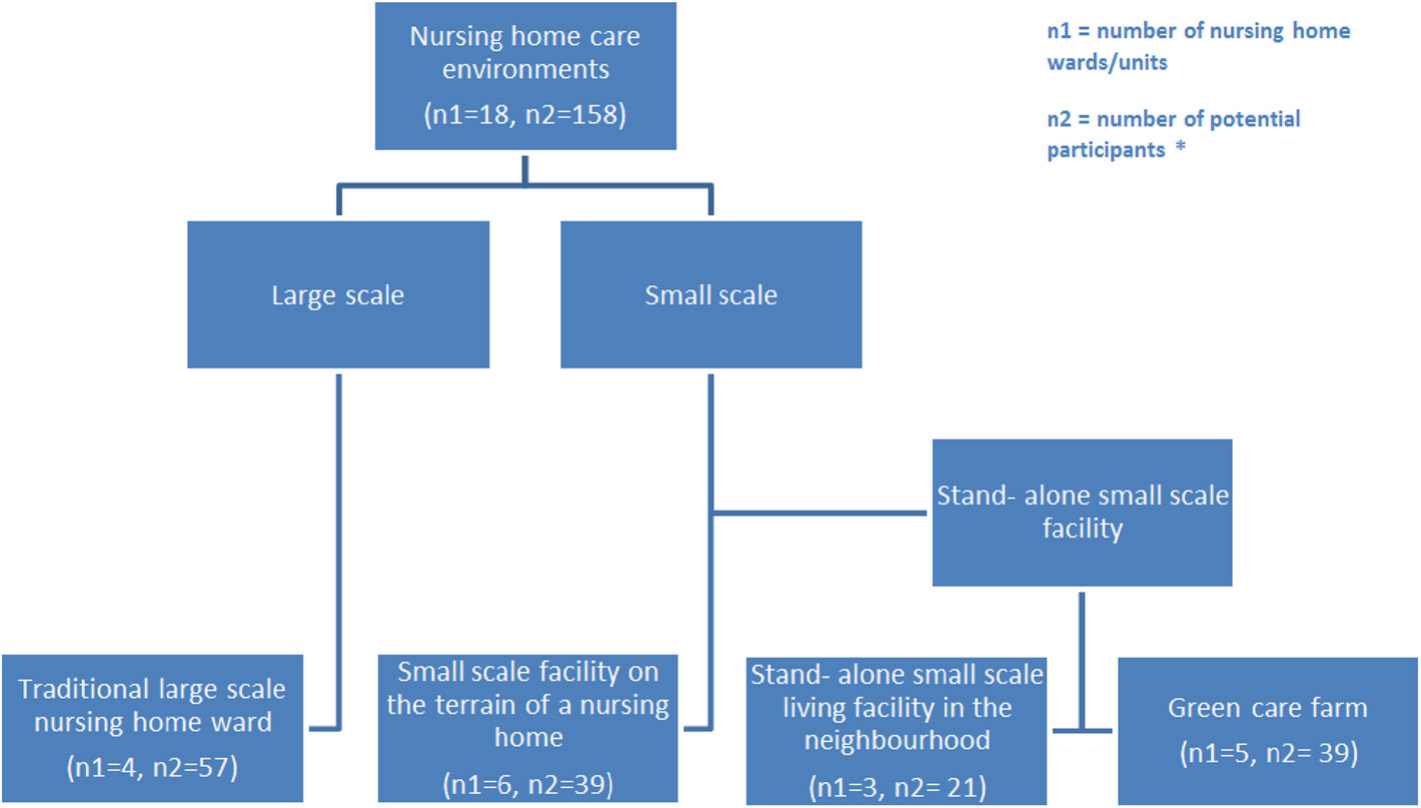
Overview of the different types of nursing home environments, the number of wards and the number of potential participants for this study. *Have a formal diagnosis of dementia

Participants of this study are all admitted to or working in non-profit, collectively funded nursing homes in the southern part of the Netherlands. In order to be admitted to these facilities, the level of care people with dementia need is determined by a standardized procedure carried out by a governmental agency. Based on this procedure, determination regarding admission to a nursing home environment is made. Both large- and small-scale nursing home environments are compared. Figure 1 gives an overview of the different types of nursing home environments, the number of wards and the number of potential participants for this study.

Four types of nursing home environments are included:

- Large-scale nursing home ward: has at least 20 residents on the ward; caregivers have differentiated tasks, aimed at nursing home care and daily life is determined by the routines and rules of the organization [12].
- Small-scale living facility on the terrain of a larger nursing home: has a maximum of 8 residents; has a joint household in which every day there is cooking in the home; caregivers have integrated tasks (they have multiple functions next to their care function); has a steady team of caregivers; daily living is mainly determined by the residents and informal caregivers; and the physical environment approaches a home like situation as much as possible [12]. In addition, residents and staff have access to facilities provided by a large nursing home facility such as a restaurant and activity areas.
- Stand- alone small scale living facility: has the same characteristics as a small scale living facility on the terrain of a larger nursing home, however, it is situated in a neighborhood and therefore does not have direct access to facilities provided at a larger nursing home. This facility is aimed at keeping contact with the community and opportunities to maintain a social network [12].
- Green care farm: A type of stand-alone small-scale nursing home facility where both care and agricultural activities are important [18]. Approximately 8 residents live together in a house on the area of the farm. Some of these green care farms are actual farms that have agricultural production while, for others, providing care is the main source of income and gaining agricultural production is a byproduct [24].

### Participants

#### Residents

All participants of this study receive a similar degree of nursing home care. Residents are eligible for participation in the study if they have a formal diagnosis of dementia according to their medical record.

Previous studies showed that residents living in small-scale facilities had a significantly better functional status and cognitive performance than residents living in large-scale facilities [9, 34]. Following earlier research [8], to prevent large differences between participants at baseline on cognition and functional dependence, a matching procedure is carried out two weeks before the baseline measurement. This increases the comparability between the participants of the different nursing home environments. The Minimum Data Set (MDS) subscales for cognition (CPS) and ADL (ADL-H) are used to screen all residents [35–37]. Residents of large-scale nursing home wards who have more or less similar scores as residents of the small scale facilities are invited to participate.

#### Caregivers

In order to explore the experiences of caregivers, a convenient sample of formal and informal caregivers is interviewed up to the point of saturation. Caregivers of all four types of nursing home care environments are interviewed.

### Measures

Table 1 summarizes the outcomes and outcome measures included in this study. Measurement instruments are selected based on their psychometric properties, the appropriateness for the target population and their availability in Dutch.

**Table 1:** Variables, operationalization and measures of the study

Variable type	Operationalization	Measure
Matching	Cognition	Cognitive Performance Scale [36]
	Activities of daily living (ADL)	ADL-Hierarchy Scale [37]
Quality of care outcomes	Structure indicators: number of residents and caregivers; total amount of hours worked; educational level of caregivers; competences of caregivers	Documentation care facility + semi-structured interviews formal caregiver
	Process indicators: presence of protocols patient safety; accessibility of protocols; the way protocols are followed	
	Outcome indicators: falling incidents; pressure ulcers; malnutrition; use of psychotropic drugs; use of restraints	Questionnaire (quality framework responsible care)
Primary outcome measure	Daily life	Maastricht Electronic Daily Life Observation tool (MEDLO-tool) [38]
Secondary outcome measures	Social engagement	Revised Index for Social Engagement (RISE) [40]
	Quality of life	Quality of Life- Alzheimer’s Disease (QoL-AD) [41]
		QUALIDEM [43]
	Behavioral symptoms	Neuropsychiatric Inventory – Nursing Home Version (NPI-NH) [45]
	Agitation	Cohen Mansfield Agitation Inventory (CMAI) [47]
Additional variables	Demographics	Medical record
	Comorbidity	Medical record
	Cognitive impairment	Standardized Mini Mental State Examination (S-MMSE) [49, 50]
	Activities of daily living (ADL)	Barthel index [51, 52]
	Severity of dementia	Global Deterioration Scale (GDS) [53]
	Environmental characteristics	Checklist
Experiences formal and informal caregivers	Formal: a specific description of a ‘typical’ day, necessary competences, required skills, attitude and knowledge, training/schooling, pros and cons of working within a particular care environment, work-related pressure and the quality of care in general.	Semi-structured interviews
	Informal: the process of choosing a particular care setting, expectations regarding the quality of care, the general perspective on quality of care, positive and negative experiences with the care facility and points of improvement.	

#### Daily lives of residents

The primary outcome measure of the study is the daily life of the residents. This is assessed by means of momentary assessments using the Maastricht Electronic Daily Life Observation tool (MEDLO-tool) [38]. The MEDLO- tool uses principles of ecological momentary assessment (EMA)[39], meaning that observations are carried out in the moment, within the context they occur which enables researchers to study the interaction between several aspects of daily life and contextual factors. The MEDLO- tool was specifically developed to provide a full and extensive description of the daily lives of people with dementia living in a nursing home care facility, including the following aspects:

- Activity
  - The activity performed by or occurring in the immediate environment of the resident (scored on a list of 32 possible activities)
  - The engagement in this activity (e.g., no engagement, passively engaged or actively engaged)
  - The degree of physical effort during this activity (ranging from lying or sitting without movement to whole-body movement)
- Physical environment
  - The location of the resident (e.g., in the communal area, own room or outside)
  - Whether the resident had interaction with the physical environment (yes or no)
- Social environment
  - The level of social interaction (ranging from no social interaction to interaction with two or more people)
  - The type of social interaction (e.g., positive social or negative restrictive)
  - With whom this social interaction takes place (e.g., family, other resident or staff)
- Psychological well-being
  - Mood (e.g. small signs of negative mood or considerable signs of positive mood)
  - Agitation (ranging from no agitation to extreme agitation)

Each aspect of daily life is observed and scored using standardized scoring options. A pilot study demonstrated that agreement levels on the domains of the MEDLO-tool are high with an average absolute agreement of 86 %. More details regarding the MEDLO- tool will be published elsewhere and are available upon request.

#### Secondary outcome measures

##### Social engagement

Social engagement is measured using the Revised Index for Social Engagement (RISE) for long-term care [40]. The RISE consists of 6 dichotomous items that measure positive features of long-term care residents’ social behavior leading to a score between 0 (lowest social engagement) and 6 (highest social engagement). The RISE has a Cronbach’s alpha coefficient of .73, and an intra-class coefficient of .75 [40].

##### Quality of life

Quality of life is measured using two questionnaires often used in dementia care research. The Quality of Life- Alzheimer’s Disease (QoL-AD)[41] provides an overall quality of life (QoL) score by means of thirteen QoL domains rated on a four-point Likert scale, ranging from 1 (poor) to 4 (excellent). Total scores range from 13 to 52, and higher scores indicate a better QoL. The QoL-AD has been found to have good content and construct validity and has a Cronbach’s alpha ranging from .82 to .90 [41, 42].

The QUALIDEM [43] is a multi-dimensional scale consisting of 37 items across 9 subscales. The subscales are care relationship (7 items), positive affect (6), negative affect (3), restless tense behavior (3), positive self-image (3), social relations (6), social isolation (3), feeling at home (4) and having something to do (2). Items are rated using four response options: never, seldom, sometimes and often. The QUALIDEM was found to have good validity [43], the Cronbach’s alpha of the subscales of the QUALIDEM are .59 for social isolation, .64 for positive self-image, .71 for negative affect, .73 for feeling at home, .74 for restless tense behavior, .80 for social relations, .83 for care relationship and .89 for positive affect [44].

##### Behavioral symptoms

The Neuropsychiatric Inventory – Nursing Home version (NPI-NH) is used to measure behavioral symptoms [45, 46]. This instrument evaluates behavioral disturbances in people with dementia. It includes 12 neuropsychiatric symptoms (domains): 1) delusions; 2) hallucinations; 3) agitation; 4) depression/dysphoria; 5) anxiety; 6) euphoria/elation; 7) apathy/indifference; 8) disinhibition; 9) irritability/lability; 10) aberrant motor behavior; 11) nighttime disturbances; and 12) appetite/eating change. First, the presence of the symptoms is scored (yes/no). Second, the frequency of the symptoms is scored as rarely (1), sometimes (2), often (3), or very often (4). Third, the severity of the symptoms is scored as mild (1), moderate (2), or severe (3). The score for each domain is calculated by multiplying the frequency and severity. One study reports a Cronbach’s alpha of 0.67 for the NPI-NH and convergent and discriminant validity were considered satisfactory [45].

##### Agitation

The frequency of agitated behaviors is measured with the Cohen Mansfield Agitation Inventory (CMAI) [47]. The inventory consists of 29 items being scored on a 7-point scale of frequency (ranging from 1 = never to 7 = several times an hour). Total scores can be calculated ranging from 29 to 203 with higher scores indicating a higher frequency of agitated behaviors. Previous studies demonstrated that the CMAI was found to be a valid measure of agitation for nursing home residents; the Cronbach’s alpha was .86 [48].

##### Additional variables

The Standardized Mini-Mental State Examination (S-MMSE) [49, 50] is used to assess cognitive impairment. Scores on the S-MMSE range from 0 to 30, with higher scores indicating better cognition. The (in) dependence in activities of daily living (ADL) is assessed with the Barthel index [51, 52]. The Barthel index consists of 10 items and has a range of 0–20, with higher scores indicating less ADL dependence. The Global Deterioration Scale (GDS) [53] is used to measure the severity of the dementia on a range from 1 (normal) to 7 (highly severe dementia). Residents’ medical records are used in order to retrieve background information regarding age, gender, admission date, type of dementia and comorbidities. Environmental characteristics of the different settings in the study are assessed using an observation checklist based on previous research [14, 54, 55]. The checklist is specifically developed to measure long term care environments in a Dutch setting. It consists of 72 items which assess aspects of the environment on a 5-point Likert scale, ranging from 1 ‘not at all’ to 5 ‘completely’. The checklist is divided over 7 themes: 1) privacy and autonomy, 2) sensory stimulation, 3) view and nature, 4) facilities, 5) orientation and routing, 6) domesticity and 7) safety. Higher scores indicate a higher probability for the environment to have a positive effect on its’ residents. An example item is: ‘there is enough space for the resident to receive visitors in his/her own room’.

#### Quality of care

To examine quality of care the model of Donabedian is used [56]. This is a widely recognized framework that divides quality of care in structure, process and outcome indicators.

- Structure indicators refer to all organizational aspects of providing care [56]. In this study the following indicators are assessed: the number of residents and caregivers, the total amount of hours worked by nursing staff within a care facility, the staff/patient ratio, the educational level of the caregivers and the competences they need when providing care.
- Process indicators refer to the way care is provided [56]. The current study assesses the presence of protocols regarding patient safety, the accessibility of these protocols and the way these protocols are implemented. Information regarding the structure and process indicators are gathered through documents provided by the nursing homes and through semi-structured interviews with caregivers.
- Outcome indicators refer to consequences of care [56]. In this study falling incidents, pressure ulcers, malnutrition and the use of psychotropic drugs or restraints are assessed. Outcome indicators are assessed with questionnaires based on the quality framework responsible care in the Netherlands [57].

#### Experiences caregivers

##### Formal caregivers

Within the four types of nursing home care environments semi-structured interviews with nursing staff are carried out. Topics discussed during these interviews include: a specific description of a ‘typical’ day at the care facility, competences necessary to provide care within a certain type of nursing home care facility, required skills, attitude and knowledge, training/schooling, differences between types of nursing home care environments in terms of pros and cons of working within a particular type, work-related pressure and the quality of care in general.

##### Informal caregivers

Semi-structured interviews are also carried out with informal caregivers. The goal is to interview the family member of the resident that is the most involved in the care of the resident. Topics discussed during these interviews include: the process of choosing a particular care setting, expectations regarding the quality of care, the general perspective on quality of care, positive and negative experiences with the care facility and points of improvement.

### Procedure

**Fig. 2:**
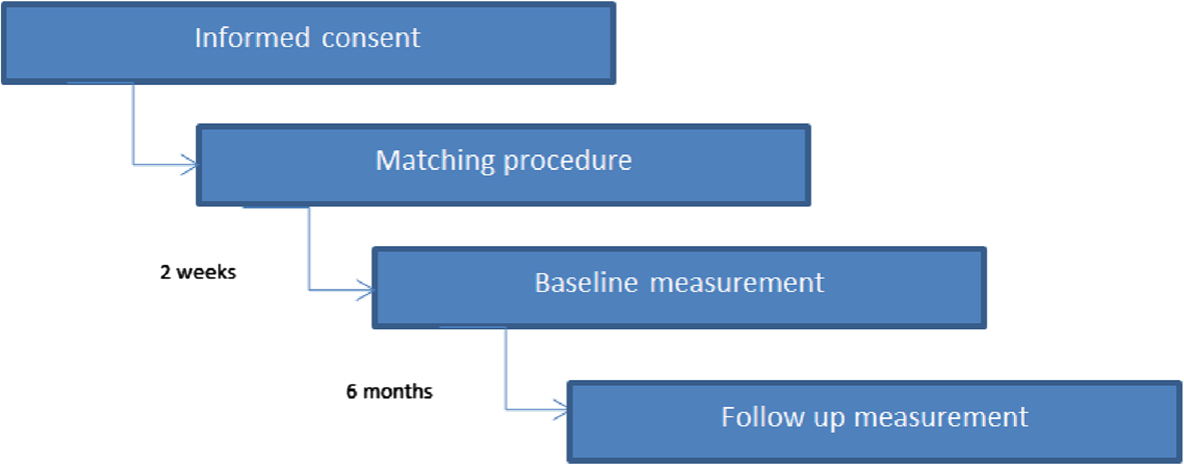
Procedure of the study

Figure 2 shows the procedure of the study. After all participating nursing homes agreed to participate in the study; written consent is obtained through the legal representatives of the residents. Second, approximately two weeks before the baseline measurement, the cognitive performance scale and the ADL- hierarchy scale are filled out for all residents in order to match residents at baseline. Third, all measures using questionnaires and documents of the care facilities are collected through certified nursing assistants who provide hands-on care to the residents of the participating care facilities at both the baseline and the follow-up measurement. The S-MMSE and the QoL-AD are administered with the residents. Quality of care outcomes and the interviews with the formal and informal caregivers are only carried out at baseline.

Data on the daily lives of the residents is collected through observations with the MEDLO-tool. Tablets are used to record momentary assessments of the different aspects of daily life. The observations take place on 2 mornings (7:00 AM-11:30 AM), 2 afternoons (11:30 AM-16:00 PM), and 2 evenings (16:00 PM-20:30 PM) at baseline and follow-up to reliably represent an ordinary day. In addition, one Saturday afternoon is included at baseline as the weekend may be different from the week-day. Thus, data was collected on 7 observation days. If it turns out there are no differences in the daily lives during the weekends, the Saturday will be excluded at the follow up measurement. Each observation day includes a half hour break for the observer, meaning that residents are observed for four hours each observation day. Every 20 min a maximum of eight residents are observed in a random order leading to 12 observations per resident each observation day. Eventually, this leads to a minimal of 156 observations per resident.

For each observation, the aspects of daily life are observed. In addition, the observer has the option to make field notes during the observations; this is done in case events occurred that could not be covered by the aspects of daily life from the MEDLO-tool. These field notes can be about the organizational, social, or physical environment.

Formal and informal caregivers receive an information letter about the study. After giving consent to participate in the study they are contacted via telephone or email for planning a date and location for an interview. The interviews are recorded with a recording device. After the interview, the recordings will be transcribed into a written transcript. The transcripts will be double checked and if necessary a member check will be carried out.

### Ethics

The medical ethics committee of the Maastricht University Medical Center reviewed the study; they declared that the study was non-invasive for people with dementia according to the Medical Research Involving Human Subjects Act [58]. All participating nursing homes provided informed consent. Legal representatives of the residents are approached for written informed consent. In addition, the residents might not always have a full understanding of the study or may not be able to sign informed consent. Therefore, they are asked to assent [59], which is defined as willingness to participate even without full understanding of the complexity and the whole aims of the study. Formal and informal caregivers are always asked permission to record the interviews.

### Statistical analysis

The data will be analyzed using IBM SPSS Statistics version 22 [60]. For each variable, descriptive statistics at both baseline and follow-up measurement and the differences between them are computed for each type of nursing home care environment separately. Comparisons of residents’ characteristics, and primary and secondary outcomes, are made between the four types of nursing home care environments. Regarding the observations of daily life; percentages will be calculated to investigate the proportion of time residents spend in each activity being scored. In addition, it is investigated whether or not residents are engaged in these activities, where residents spent their time, whether or not they have social interaction and with whom, how much physical effort they need during their days and their average mood and agitation levels are calculated. Furthermore, mixed-effects regression analyses are used to estimate differences between the types of nursing home care environments, taking into account that the momentary assessments (observations) on daily life are nested within participants.

Analyses of the interviews are done by several coding steps [61]. Open coding is used to discover concepts discussed during the interviews, these are called ‘main themes’. These main themes are subcategorized and by axial coding related to each other. The last step is selective coding; this is the process of integrating and refining categories. The whole coding process will be done by two researchers independently.

## Discussion

To our best knowledge, this is the first study that investigates green care farms that provide 24-h nursing home care for people with dementia. The aim of this study is to explore the daily lives of residents at green care farms in comparison with other nursing home care environments. Furthermore, the quality of care, and the experiences of caregivers with the care environment are assessed. Green care farms are compared with other nursing home care environments (both large- and small-scale). The combination of momentary assessments and the use of questionnaires provide in-depth knowledge about important outcome measures for residents. The number of momentary assessments per resident gives a more precise estimate of the activities of residents, their quality of life, and physical and psychological well-being than a single measurement. It offers the possibility to investigate the added value of green care farms on the spectrum of nursing home care environments.

Due to ethical considerations, it is impossible to randomly allocate participants to a certain type of nursing home care environment. Therefore, it is possible that a certain nursing home care environment attracts a specific type of resident. For example, previous research has shown that residents in small-sale, homelike care environments have better cognitive and functional abilities compared with residents in traditional large-scale nursing home wards [9]. In order to prevent these differences and to increase comparability of residents at baseline, participants in the current study are matched on cognition and ADL- capacity.
